# Hematologists’ awareness of venous thromboembolism in multiple myeloma: a national survey in China

**DOI:** 10.1080/07853890.2023.2263019

**Published:** 2023-11-20

**Authors:** Qun Li, Bo Zhang, Qianwen Cheng, Fei Zhao, Junying Li, Han Yan, Aoshuang Xu, Chunyan Sun, Yu Hu

**Affiliations:** Institute of Hematology, Union Hospital, Tongji Medical College, Huazhong University of Science and Technology, Wuhan, China

**Keywords:** Venous thromboembolism, multiple myeloma, risk assessment, stratified thromboprophylaxis

## Abstract

**Background:**

Venous thromboembolism (VTE) is one of the most common and severe complications of multiple myeloma (MM). The aim of this study was to learn about the current awareness regarding MM-associated VTE among Chinese hematologists.

**Methods:**

A nationwide, online, questionnaire-based survey was sent to the specialized hematologists in mainland China. The questionnaire investigated respondents’ demographic and occupational characteristics, their ability to identify VTE risk factors, and their thromboprophylaxis decisions for different anti-MM regimens. Six clinical vignettes were used to evaluate hematologists’ awareness of stratified thromboprophylaxis. The data were analyzed using SPSS software.

**Results:**

A total of 518 valid questionnaires were received. Of the 518 hematologists investigated, only 23.7% of them could identify VTE-related risk factors correctly. Most hematologists could select appropriate thromboprophylaxis for common anti-MM regimens such as VCd (bortezomib, cyclophosphamide, and dexamethasone) and VRd (bortezomib, lenalidomide, and dexamethasone), but not for uncommon ones such as VTD-PACE (bortezomib, thalidomide, dexamethasone, cisplatin, doxorubicin, cyclophosphamide, and etoposide) and KRd (carfilzomib, lenalidomide, and dexamethasone). The results from the vignettes suggested that only 19.5% of the hematologists could be defined as the ‘stratified thromboprophylaxis’ group, and the awareness of stratified thromboprophylaxis depended significantly on physicians’ age and working seniority.

**Conclusion:**

The results of our study showed that a large proportion of Chinese hematologists failed to recognize the VTE risk factors, most of them cannot select appropriate thromboprophylaxis for different MM therapeutic regimens and lack awareness of stratified thromboprophylaxis for MM-associated VTE. A standard VTE prevention guideline is urgently needed for the Chinese myeloma group. Continuous education for new professionals should be encouraged. A VTE collaborative group is supposed to be established in each hospital to enhance the overall medical care for VTE patients.

## KEY MESSAGES

- The substantial myeloma patient population and extensive use of immunomodulatory drugs indicate that MM-associated VTE deserves more attention in China.
- The results from our cross-sectional study showed that a significant proportion of Chinese hematologists failed to identify the VTE risk factors, most of them cannot select appropriate thromboprophylaxis for different MM therapeutic regimens and lack awareness of stratified thromboprophylaxis for MM-associated VTE.

## Introduction

Venous thromboembolism (VTE) is one of the most common and dangerous complications of Multiple myeloma (MM). It occurs in more than 10% of MM cases and is associated with increased mortality [1,2]. There are three major causes of VTE occurrence: patient-related factors such as advanced age and immobility; myeloma-related factors such as disease burden and hyperviscosity; and what calls for special attention are the treatment-related factors [3]. Among the available drugs approved for MM treatment, immunomodulatory drugs (IMiDs), including lenalidomide and thalidomide, seemed to contribute significantly to the development of VTE when combined with dexamethasone or doxorubicin-based chemotherapy [4]. Proteasome inhibitors like bortezomib and ixazomib showed a relatively low risk of VTE [5,6]. However, the second-generation proteasome inhibitors, carfilzomib, appeared to be more thrombogenic due to its toxicity to vascular endothelial cells [7]. Given the adverse impacts of VTE, including treatment interruption, a higher death rate, and an economic burden, the accurate identification of VTE risk factors and the appropriate application of thromboprophylaxis are of great importance.

Several attempts in the field of prophylaxis for MM-associated VTE have been made in the past few years. Two early randomized prospective trials tried to compare aspirin, warfarin, and low molecular weight heparin (LMWH) for VTE prevention in myeloma patients who received IMiDs-based therapy. However, patients at high risk of thromboembolism were excluded from both studies [8,9]. In 2014, the International Myeloma Working Group (IMWG) recommended risk-stratified thromboprophylaxis for MM-associated VTE on the basis of restricted data and expert opinions [3]. As compared with that only 22.3% of patients received thromboprophylaxis without the IMWG guideline and the VTE rate was 15.2% in the Myeloma IX trial, the rate of VTE reported from the Myeloma XI trial was still unacceptablely high (12.2%) despite 80.5% of patients receiving thromboprophylaxis according to the IMWG guideline, which indicated the limited power of the IMWG model [10]. Two novel risk assessment models, the IMPEDE VTE and SAVED scoring systems, have been developed retrospectively. The IMPEDE VTE system included risk scoring based on the **i**mmunomodulatory agent; body **m**ass index ≥ 25 kg/m^2^; **p**elvic, hip, or femur fracture; **e**rythropoietin stimulating agent; **d**examethasone/doxorubicin; Asian **e**thnicity/race; **V**TE history; **t**unneled line/central venous catheter; and **e**xisting thromboprophylaxis [8]. The SAVED score consisted of 5 clinical variables: prior **s**urgery, **A**sian race, **V**TE history, **a**ge ≥ 80 years, and **d**examethasone dose [9]. Both models were proved to outperform the IMWG guideline with c-indices greater than or equal to 0.6 [8,9]. Rencently, some research groups proposed that in addition to the ‘low-risk’ and ‘high-risk’ groups, an extra ‘very high-risk’ group should be added into the assessment systems to fully protect patients who were at the highest thrombosis risk [4,11]. However, solid evidence-based recommendations were still lacking despite these efforts, and the management of MM-associated VTE in the real world was still far from satisfactory [10,12].

Asian–Pacific Islanders have a reduced risk of developing thromboembolism [13,14]. As a result, hematologists in many Asian centers might not pay enough attention to the occurrence of VTE [15,16]. However, MM-associated VTE in China could be a significant problem because of the increased patient population and the extensive use of IMiDs. In the present study, a cross-sectional survey was conducted among Chinese hematologists to investigate the current awareness of MM-associated VTE. Our study aimed to draw national attention to MM-associated VTE and promote the overall management of MM patients in China.

## Materials and methods

### Study design

This cross-sectional national study was carried out from April 26 to May 22, 2022. Since it is challenging to identify the total number of registered hematologists practicing in the country, we performed the survey *via* the online WeChat platform. The questionnaire link was distributed to a number of national hematology specialists *via* WeChat groups, and then forwarded to the hematologist groups in their provinces and cities. To completely understand the current awareness of MM-related VTE in China, hematologists from multiple levels of hospitals (such as township hospital, county hospital, municipal hospital, provincial or ministerial hospital) with different professional titles, including resident physician, attending physician, associate chief physician, and chief physician, were all included in the survey. Participation was voluntary, and informed consent was implied upon completing the survey.

### Questionnaire

A draft questionnaire was developed and modified after pilot testing with 20 hematologists. The final version consisted of 36 questions and took 10–15 min to finish. The complete questionnaire is provided in Supplementary file 1. Overall, the questionnaire contained four parts. The first part collected information on the respondents’ demographic and occupational characteristics (including gender, age, hospital level, working seniority, hierarchical position, and number of MM patients they treat annually). It also gathered data on their prior experiences with the application of VTE risk assessment and stratified thromboprophylaxis for MM patients.

The second part aimed to ascertain hematologists’ capacity to identify VTE risk factors with varying weights. A series of VTE risk factors were provided in an arbitrary sequence in Question#19, and the participants were asked to evaluate the weight of each factor in VTE occurrence on a scale from 0 to 3. We summarized these factors into four categories (A–D) for data analysis according to the published VTE risk assessment recommendations and defined that the order of the categories represented a progressive prothrombotic power. The detailed categorization is presented in Supplementary Table 1 [3,8,9,17–20]. We defined that participants who got an ascending average score from category A to category D were classified into the ‘capable of identifying the VTE risk factors’ group.

The third part listed a variety of anti-MM chemotherapy regimens. There were five prophylaxis choices given when each regimen was used for MM patients in the absence of any additional VTE risk factors: aspirin, LMWH, rivaroxaban, warfarin, or no thromboprophylaxis. This section looked into how hematologists perceived the treatment-related VTE risk factors and what thromboprophylaxis decisions they made for different regimens. We evaluated the results in light of the existing guidelines and prior researches [3,7,21–27].

In the fourth section, six clinical vignettes were presented in order to assess hematologists’ awareness of risk-stratified thromboprophylaxis. These vignettes were derived from real clinical cases and summarized by two experts. Cases in vignettes 1–3 were thought to be low-risk for VTE. Patients in vignettes 4–6 appeared to have thrombogenic risk factors such as immobility, a pathological fracture, a history of thrombosis, or a peripherally inserted central catheter (PICC). These patients could be at a high or extremely high risk of developing VTE when receiving IMiDs-based therapy [3,8,9,17–20]. Each vignette was given seven options, and each option was assigned a score: (1) No thromboprophylaxis (0 point); (2) Aspirin (1 point); (3) Warfarin (INR 2.0–3.0, 2 points); (4) LMWH at a prophylactic dose (such as enoxaparin at 40 mg/d, 2 points); (5) LMWH at a therapeutic dose (such as enoxaparin at 1 mg/kg, twice daily, 3 points); (6) Direct oral anticoagulants (DOACs) at a prophylactic doses (such as rivaroxaban at 10 mg/d; 2 points); (7) DOACs at a therapeutic dose (such as rivaroxaban at 20 mg/d; 3 points). Under the guidance of the current VTE score systems and guidelines [3,8,9,17–21], we conducted an analysis of the responses. The hematologists were split into two clusters based on the thromboprophylaxis selection for the six vignettes. Those who got the sum of scores ≤3 for vignettes 1–3 and ≥6 for vignettes 4–6 at the same time were defined as the ‘stratified thromboprophylaxis’ group, while the others were defined as the ‘non-stratified thromboprophylaxis’ group.

The collected questionnaires were subject to quality control, responses that were not complete, came from the same IP, or took less than five minutes to finish were deemed invalid.

### Statistical analysis

The qualitative data were presented as counts and frequencies. The *χ*^2^-test was used to compare the categorical variables between the two groups. A two-sided *p* < .05 was considered statistically significant. All analyses were computed using SPSS statistics V.22.0 (SPSS Inc., Chicago, IL).

## Results

### Demographic and occupational characteristics

Overall, a total of 518 valid questionnaires were received. Respondents were distributed in 29 provinces and municipalities, 200 (38.6%) of them were men, 342 (66.0%) were 40 years old or above, and 232 (44.8%) had more than 20 years of job experience. Most of them worked in provincial or ministerial hospitals (236, 45.6%) and municipal hospitals (247, 47.7%). 152 participants (29.3%) had junior titles like resident and attending physician, whereas the remaining had advanced titles like associate chief and chief physician (366, 70.7%). 159 (30.7%) of them treated more than 40 MM patients each year. The detailed demographic and occupational characteristics are shown in Table 1.

**Table 1.** Baseline comparison between hematologists who were capable and incapable of identifying the VTE risk factors.

	Overall sample	Incapable group	Capable group	
	(*n* = 518)	(*n* = 395)	(*n* = 123)	
Variable	*N* (%)	*N* (%)	*N* (%)	*p* value
Gender				.92
Men	200 (38.6)	153 (38.7)	47 (38.2)	
Women	318 (61.4)	242 (61.3)	76 (61.8)	
Age, years				.09
<40	176 (34.0)	142 (35.9)	34 (27.6)	
≥40	342 (66.0)	253 (64.1)	89 (72.4)	
Working hospital level				.96
* Provincial* or ministerial hospitals	236 (45.6)	180 (45.6)	56 (45.5)	
Municipal hospitals	247 (47.7)	189 (47.8)	58 (47.2)	
County or township hospitals	35 (6.8)	26 (6.6)	9 (7.3)	
Working seniority, years				.55
≤20	286 (55.2)	221 (55.9)	65 (52.8)	
>20	232 (44.8)	174 (44.1)	58 (47.2)	
Positional titles				.04*
Resident and attending physician	152 (29.3)	125 (31.6)	27 (22.0)	
Associate chief and chief physician	366 (70.7)	270 (68.4)	96 (78.0)	
Number of patients with MM admitted annually				.54
≤40	359 (69.3)	271 (68.6)	88 (71.5)	
>40	159 (30.7)	124 (31.4)	35 (28.5)	
Did you assess the risk of VTE for MM patients?				.62
No	209 (40.3)	157 (39.7)	52 (42.3)	
Yes	309 (59.7)	238 (60.3)	71 (57.7)	
Did you carry out stratified thromboprophylaxis for MM patients?				.11
No	74 (14.3)	51 (12.9)	23 (18.7)	
Yes	444 (85.7)	344 (87.1)	100 (81.3)	

*χ*^2^-test was used to compare categorical variables.

VTE: Venous thromboembolism; MM: Multiple Myeloma. Bold emphasis and asterisk (*) indicate statistical significance (*p* < .05).

Over half of the hematologists involved in our study claimed that they had done risk assessment (309, 59.7%) for patients with MM, but only a minority of them used the IMPEDE VTE Score (25, 4.8%) or SAVED Score (11, 2.1%) (Supplementary Figure 1). 444 (85.7%) of them claimed they have carried out stratified thromboprophylaxis, but mostly according to self-made guidelines (194, 37.5%) or clinical experience (171, 33.0%) (Supplementary Figure 2).

### Identification of the VTE risk factors

123 respondents (23.7%) who got an ascending average score from category A to category D on question 19 were classified as the ‘capable of identifying the VTE risk factors’ group, while the remaining 395 people (76.3%) were categorized as the ‘incapable’ group. Compared with the hematologists in the ‘incapable’ group, those in the ‘capable’ group possessed higher-level of professional titles (*p* = .04). Statistics were not significantly different between the two groups in terms of other characteristics (Table 1).

### Thromboprophylaxis decision-making for various anti-MM regimens

**Figure 1.**
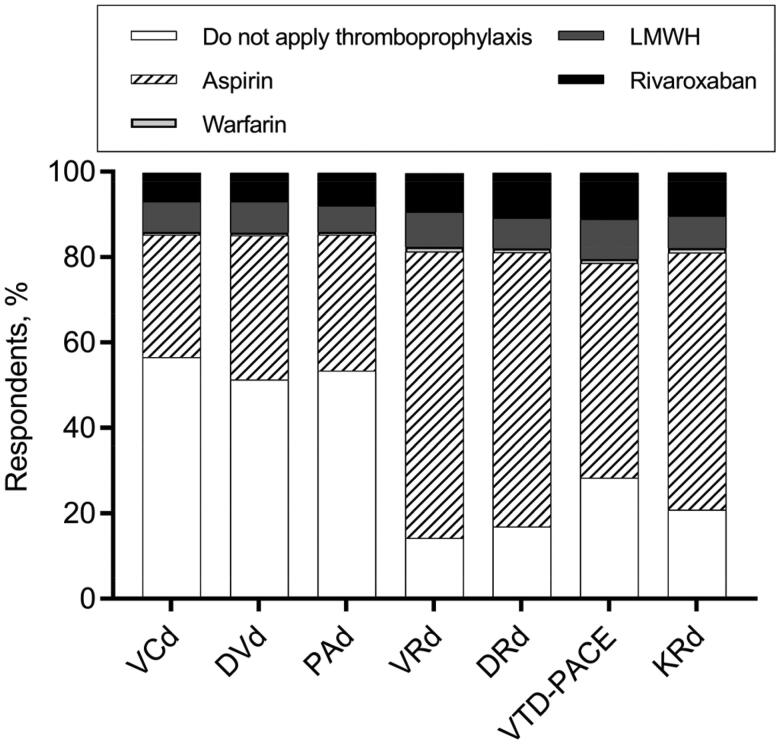
Decision-making of thromboprophylaxis for various anti-MM regimens. VCd: bortezomib; cyclophosphamide, and dexamethasone; DVd: daratumumab, bortezomib, and dexamethasone; PAd: bortezomib, doxorubicin, and dexamethasone; VRd: bortezomib, lenalidomide, and dexamethasone; DRd: daratumumab, lenalidomide, and dexamethasone; VTD-PACE: bortezomib, thalidomide, dexamethasone, cisplatin, doxorubicin, cyclophosphamide, and etoposide; KRd: carfilzomib, lenalidomide, and dexamethasone.

In our study, more than 60% of the respondents decided to prescribe aspirin for patients who were treated with VRd (bortezomib, lenalidomide, and dexamethasone) or DRd (daratumumab, lenalidomide, and dexamethasone) without any extra risk factors for VTE (Figure 1), which was in accordance with the guidelines’ suggestion [3,21]. Over 20% of clinicians believed that thromboprophylaxis was unnecessary for patients who received VTD-PACE (bortezomib, thalidomide, dexamethasone, cisplatin, doxorubicin, cyclophosphamide, and etoposide) and KRd (carfilzomib, lenalidomide, and dexamethasone) therapy, while more than 50% gave aspirin first priority (Figure 1). Approximately half of the hematologists decided not to prescribe pharmaceutical thromboprophylaxis for some regimens with low prothrombotic effects, such as VCd (bortezomib, cyclophosphamide, and dexamethasone) and DVd (daratumumab, bortezomib, and dexamethasone) [25,26], while the other half continued to support the use of aspirin or other stronger anticoagulants (Figure 1). A comparison of baseline characteristics between hematologists who chose thromboprophylaxis versus those who chose no thromboprophylaxis for VCd or DVd is shown in Table 2. Compared with those who refused to give thromboprophylaxis, hematologists who supported the use of thromboprophylaxis mainly worked in middle-level medical centers like municipal hospitals (*p* < .001), admitted fewer patients (*p* = .02), but claimed a higher implementation rate of stratified thromboprophylaxis (*p* = .007).

**Table 2.** Baseline comparison between hematologists who chose no thromboprophylaxis and chose thromboprophylaxis for VCd and DVd.

	Overall sample (*n* = 518)	Chose No Thromboprophylaxis Group (*n* = 240)	Chose Thromboprophylaxis Group (*n* = 278)	
Variable	*N* (%)	*N* (%)	*N* (%)	*p* value
Gender				.76
Men	200 (38.6)	91 (37.9)	109 (39.2)	
Women	318 (61.4)	149 (62.1)	169 (60.8)	
Age, years				.22
<40	176 (34.0)	75 (31.3)	101 (36.3)	
≥40	342 (66.0)	165 (68.8)	177 (63.7)	
Working hospital level				<.001*
* Provincial* or ministerial hospitals	236 (45.6)	135 (56.3)	101 (36.3)	
Municipal hospitals	247 (47.7)	96 (40.0)	151 (54.3)	
County or township hospitals	35 (6.8)	9 (3.8)	26 (9.4)	
Working seniority, years				.33
≤20	286 (55.2)	127 (52.9)	159 (57.2)	
>20	232 (44.8)	113 (47.1)	119 (42.8)	
Positional titles				.21
Resident and attending physician	152 (29.3)	64 (26.7)	88 (31.7)	
Associate chief and chief physician	366 (70.7)	176 (73.3)	190 (68.3)	
Number of patients with MM admitted annually				.02*
≤40	359 (69.3)	154 (64.2)	205 (73.7)	
>40	159 (30.7)	86 (35.8)	73 (26.3)	
Did you assess the risk of VTE for MM patients?				.14
No	209 (40.3)	105 (43.8)	104 (37.4)	
Yes	309 (59.7)	135 (56.3)	174 (62.6)	
Did you carry out stratified thromboprophylaxis for MM patients?				.007*
No	74 (14.3)	45 (18.8)	29 (10.4)	
Yes	444 (85.7)	195 (81.3)	249 (89.6)	

*χ*^2^-test was used to compare categorical variables.

MM: Multiple Myeloma; VCd: bortezomib, cyclophosphamide, and dexamethasone; DVd: Daratumumab, bortezomib, and dexamethasone. Bold emphasis and asterisk (*) indicate statistical significance (*p* < .05).

### Decision-making for some special clinical situations

**Figure 2.**
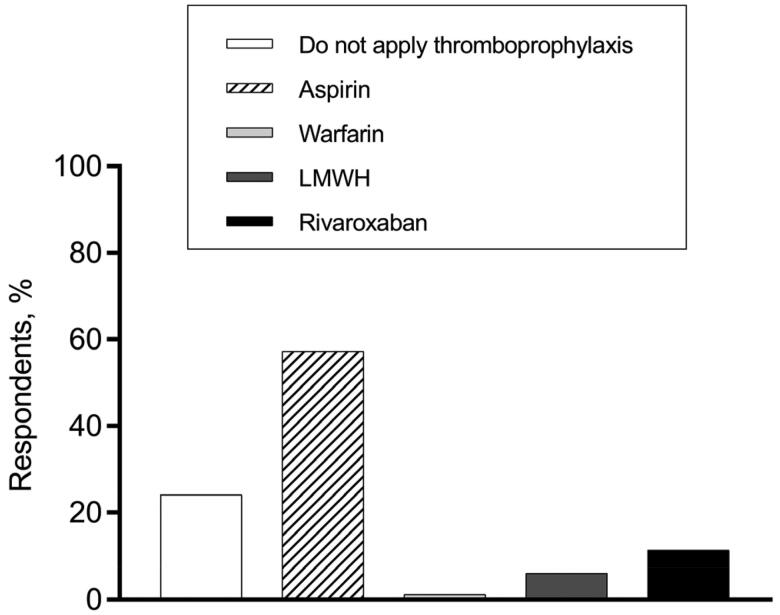
Thromboprophylaxis decisions for MM patients who have a disease relapse and receive the IMiDs-dexamethasone combined treatment.

**Figure 3.**
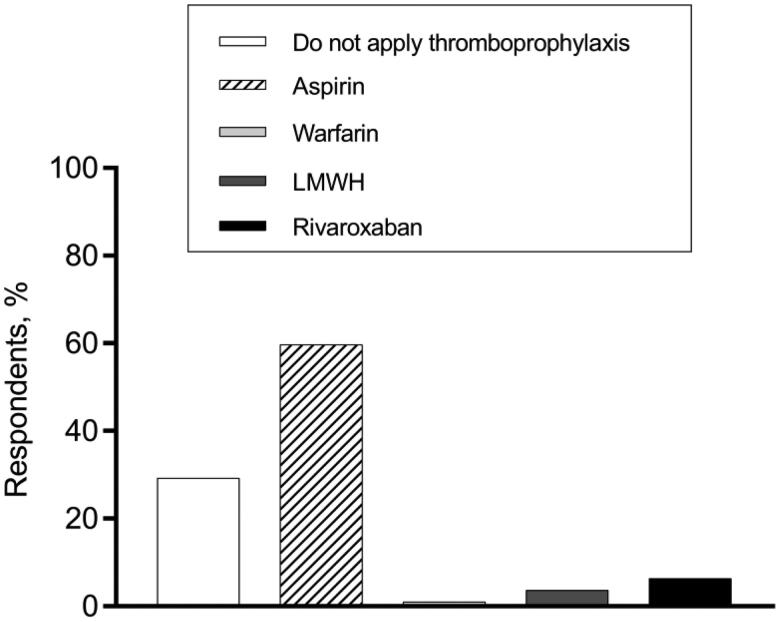
Thromboprophylaxis decisions for MM patients who receive lenalidomide for maintenance therapy.

Regarding the thromboprophylaxis choices at various phases of MM treatment, hematologists’ opinions were varied. In terms of VTE prevention for patients who had a disease relapse and received IMiDs-dexamethasone-combined treatment, 57.3% of hematologists recommended aspirin, while 24.1% supported no thromboprophylaxis (Figure 2). When it comes to thromboprophylaxis for patients who received single-agent IMiD as maintenance therapy, more than 70% of our respondents persisted in thromboprophylaxis administration, while 29.3% thought anticoagulant/antiplatelet agents were unnecessary (Figure 3).

**Figure 4.**
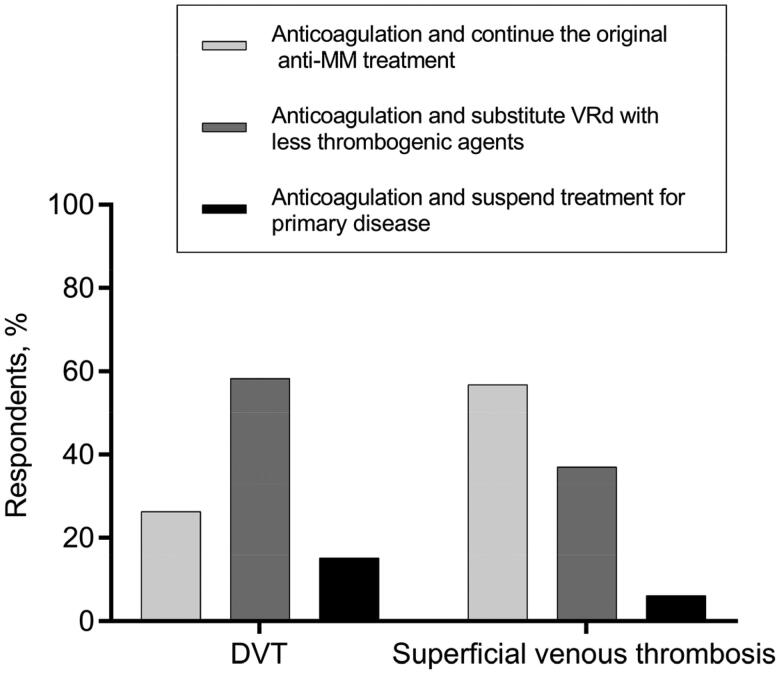
Medical decisions for MM patients who develop DVT/superficial venous thrombosis during the VRd treatment.

**Figure 5.**
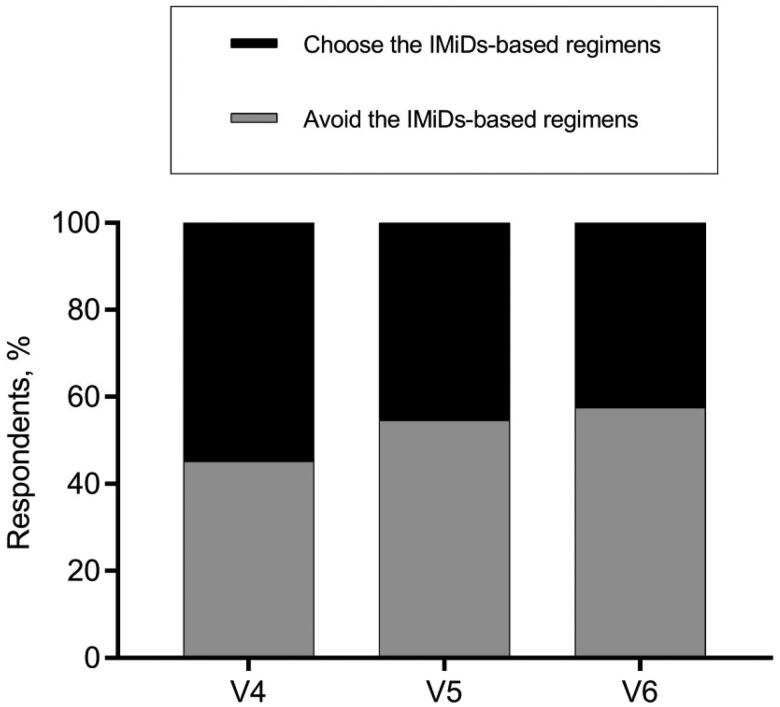
Decisions of whether or not to give IMiDs-based regimens to patients depicted in vignettes #4, #5, and #6.

Diverse opinions existed on the application of IMiDs in some complex situations as well. Regarding the decision of whether to terminate or continue the IMiDs-based regimen for MM patients who suffered a thrombosis when the primary disease was not well controlled, more than half of hematologists backed keeping the original strategy for patients who developed superficial venous thrombosis but substituting IMiDs-based regimen with less thrombogenic agents for those DVT cases (Figure 4). As for the anti-MM therapeutic decisions for patients with highly thrombotic factors, about half of the hematologists avoided prescribing the IMiDs-containing regimens for patients depicted in vignettes #4, #5, and #6, while the other half continued to give IMiDs-based treatment (Figure 5).

### Comparisons between stratified thromboprophylaxis and non-stratified thromboprophylaxis groups

**Figure 6.**
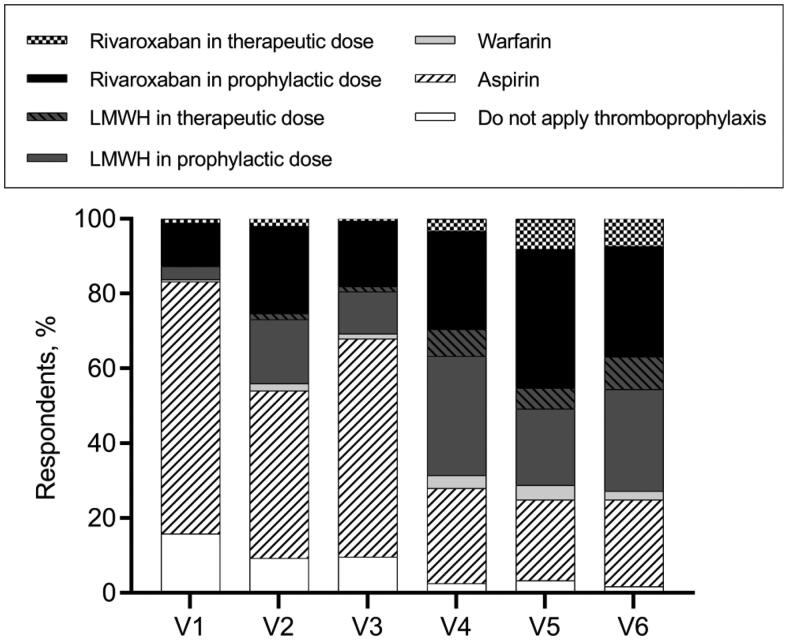
Decision-making of thromboprophylaxis for six vignettes.

The results of the six vignettes were used to assess the awareness of stratified thromboprophylaxis (Figure 6). Overall, the participants can be divided into two groups. 101 (19.5%) of the hematologists were classified into the ‘stratified thromboprophylaxis’ group, and the remaining 417 (80.5%) were classified into the ‘non-stratified thromboprophylaxis’ group. Hematologists in the ‘stratified thromboprophylaxis’ group were older (*p* = .004) and had more years of working experience (*p* = .02) compared with those in the ‘non-stratified thromboprophylaxis’ group (Table 3).

**Table 3.** Baseline comparison between hematologists in non-stratified thromboprophylaxis and stratified thromboprophylaxis groups.

	Overall sample (*n* = 518)	Non-Stratified thromboprophylaxis group (*n* = 417)	Stratified thromboprophylaxis group (*n* = 101)	
Variable	*N* (%)	*N* (%)	*N* (%)	*p* value
Gender				>.99
Men	200 (38.6)	161 (38.6)	39 (38.6)	
Women	318 (61.4)	256 (61.4)	62 (61.4)	
Age, years				.004*
<40	176 (34.0)	154 (36.9)	22 (21.8)	
≥40	342 (66.0)	263 (63.1)	79 (78.2)	
Working hospital level				.35
* Provincial or ministerial hospitals*	236 (45.6)	193 (46.3)	43 (42.6)	
Municipal hospitals	247 (47.7)	199 (47.7)	48 (47.5)	
County or township hospitals	35 (6.8)	25 (6.0)	10 (9.9)	
Working seniority, years				.02*
≤20	286 (55.2)	241 (57.8)	45 (44.6)	
>20	232 (44.8)	176 (42.2)	56 (55.4)	
Positional titles				.06
Resident and attending physician	152 (29.3)	130 (31.2)	22 (21.8)	
Associate chief and chief physician	366 (70.7)	287 (68.8)	79 (78.2)	
Number of patients with MM admitted annually				.10
≤40	359 (69.3)	282 (67.6)	77 (76.2)	
>40	159 (30.7)	135 (32.4)	24 (23.8)	
Did you assess the risk of VTE for MM patients?				.24
No	209 (40.3)	163 (39.1)	46 (45.5)	
Yes	309 (59.7)	254 (60.9)	55 (54.5)	
Did you carry out stratified thromboprophylaxis for MM patients?				.15
No	74 (14.3)	55 (13.2)	19 (18.8)	
Yes	444 (85.7)	362 (86.8)	82 (81.2)	

*χ*^2^-test was used to compare categorical variables.

VTE: Venous thromboembolism; MM: Multiple Myeloma. Bold emphasis and asterisk (*) indicate statistical significance (*p* < .05).

## Discussion

VTE poses a significant threat to the safety of MM patients. Accurate identification of the risk factors is a prerequisite for the risk assessment of VTE. Unfortunately, only 23.7% of the hematologists in our study were capable of identifying the weight of the VTE risk factor correctly. There were more hematologists with senior titles in the ‘capable’ group compared with those in the ‘incapable’ group, indicating that advanced-trained physicians may be more knowledgeable about potential thrombogenic factors than their less experienced counterparts. In addition, as evidenced by the vignettes, our respondents lacked the ability for stratified thromboprophylaxis. Less than 20% of the participants met the criteria to be classified as the ‘stratified thromboprophylaxis’ group, although 52.7% of them claimed they had applied VTE assessment according to the Caprini, Khorana or self-made scores and 70.5% of them asserted they had carried out stratified thromboprophylaxis based on their self-made guidelines or clinical experience. It revealed a fact that general VTE assessment systems such as Caprini and Khorana may not be applicable for MM-associated VTE [28] and that personal clinical experience can be undependable as well. Despite the fact that there are two risk assessment tools tailored to MM-associated VTE available (IMPEDE VTE and SAVED scores), our research suggested that neither of them has been widely adopted in China. Furthermore, we found that participants who were classified into the ‘stratified thromboprophylaxis’ group were usually 40 years old or above and had a working seniority of more than 20 years. It is reasonable to speculate that clinical experience, though not always reliable, plays a crucial role in the formation of the consciousness of VTE prevention. Junior hematologists would have trouble recognizing risk factors of VTE and would lack awareness of stratified thromboprophylaxis in their daily clinical work, which reminds us that the guidance and training for young doctors should be improved.

The results from our research indicated that of all the pharmaceutical thromboprophylaxis options, aspirin was the most appealing one. Chinese physicians’ preference for aspirin is not only because of its convenience of oral administration and absence of monitoring needs; more significantly, it may also be due to the belief that Asians are protected from VTE [29,30] and that antiplatelet medications are sufficient for patients in the absence of additional VTE risks. However, there was some evidence suggesting that aspirin may be inadequate for some extremely prothrombotic regimens, such as VTD-PACE. Zangari et al. [22] demonstrated that DVT occurred in 16% of 192 MM patients treated with DT-PACE (dexamethasone, thalidomide, cisplatin, doxorubicin, cyclophosphanide, and etoposide). Baz et al. [23] reported the highest incidence (58%) in patients receiving the DVd-T regimen (thalidomide, pegylated doxorubicin, vincristine, and dexamethasone) without pharmacological thromboprophylaxis, which was decreased by the addition of aspirin. However, there were still 18% of patients who could not be protected from the prophylaxis with antiplatelet drug [23]. Therefore, more powerful anticoagulant agents are required for individuals who received such thrombogenic anti-MM treatments. Unfortunately, the results from our research indicated that participants seemed to attach little importance to this critical issue, most of them gave aspirin first priority or no thromboprophylaxis for MM patients with the VTD-PACE regimen. What’s more, the thrombogenic capacity of the second generation proteasome inhibitor carfilzomib with toxicity to vascular endothelial cells was much more powerful than bortezomib, which can only be effectively prevented by low-dose rivaroxaban rather than aspirin [7,24]. Altogether, MM patients receiving thrombogenic drugs, including IMiDs-doxorubicin combination chemotherapy, and carfilzomib-containing regimen, may benefit from more intensive VTE prevention measures, such as DOACs, rather than aspirin. As we know, any agent for thromboprophylaxis is associated with a bleeding risk and the strategy of thromboprophylaxis should be carefully selected after balancing the risks of both VTE and bleeding. Therefore, thromboprophylaxis for some regimens with low prothrombotic effects, such as VCd and DVd, should be avoided if additional risk factors were absent [25–27]. However, most of our participants from the municipal hospitals continued to support the use of anticoagulant/antiplatelet agents. These hematologists usually admitted ≤ 40 MM patients annually and thought highly of the implementation of stratified thromboprophylaxis based on their self-evaluation. These results reflected that a correct decision was subject to not only the thromboprophylaxis awareness but also the clinical practice chance. In China, physicians from provincial or ministerial hospitals seem to have more chance to practice than those from municipal hospitals because there are more patients visiting the higher-level medical centers each year.

It was reported that relapsed/refractory MM (R/R MM) patients and newly diagnosed MM (NDMM) cases had a comparable rate of VTE [12,27]. Thus, the prophylaxis decisions for relapse treatment were supposed to be made in accordance with the indications for NDMM [27]. However, more than 20% of our participants did not recognize the significance of thromboprophylaxis for relapsed patients receiving IMiDs-dexamethasone-based therapy. As for the thromboprophylaxis strategy for maintenance therapy, over 70% of hematologists supported the use of anticoagulant/antiplatelet agents from our results. Data from the Myeloma IX trial showed that thalidomide maintenance treatment did not increase the risk of VTE. Although the VTE rate in the Myeloma XI trial was elevated in the lenalidomide maintenance group compared with that in the observation group, the absolute risk was low and far less than lenalidomide-based induction therapy. [10] Overall, the necessity of VTE prevention for IMiDs maintenance therapy is not widely accepted and is supposed to be explored in future studies. The use of IMiDs-containing regimens for MM patients who suffered a thrombosis was controversial in our study. Currently, there haven’t been any studies discussing whether or not to halt the anti-MM medication after thrombosis occurs. Considering the prothrombotic risk of IMiDs-based therapy, early thrombolytic and anticoagulant therapy along with a dose reduction of IMiDs-containing agents or the substitution of low thrombogenic regimens seems more recommended [3,4,11,21]. When it comes to the anti-MM medical decision for patients with a high risk of VTE, about half of our respondents decided to not avoid the IMiDs-containing regimens. The potential explanations for this phenomenon might be as follows: on the one hand, Chinese hematologists might give IMiDs first priority due to their availability and affordability in China, on the other hand, the application of IMiDs is not forbidden by the current guidelines when additional prothrombotic factors are present, and it might be safely used if more powerful anticoagulation measures are adopted.

This study has some limitations. First, the study population was not selected by stratified sampling, thus, it might not fully represent all hematologists in China. Besides, the congruence between hematologists’ responses to vignettes and their actual decisions made in the clinic can be doubted since patients’ preferences were not taken into account.

## Conclusion

According to the current study, we found that a significant proportion of Chinese hematologists failed to identify the VTE risk factors, most of them cannot select appropriate thromboprophylaxis for different MM therapeutic regimens and lack awareness of stratified thromboprophylaxis for MM-associated VTE. However, the substantial myeloma patient population and extensive use of IMiDs indicate that MM-associated VTE deserves more attention in China. Firstly, we expect the establishment of a national guideline for stratified thromboprophylaxis of MM-associated VTE, followed by the confirmation of its safety and efficacy in prospective randomized controlled trials. Additionally, continuous education and communication meetings with advanced-trained hematologists for new professionals and hematologists from primary hospitals should be encouraged, in light of the survey results that professionals with more experience and older ages tend to have a better understanding of VTE management in MM patients. Last but not least, given that VTE is a silent killer, it is recommended to create a VTE collaborative group in each hospital, consisting of sonographers, radiologists, hematologists, cardiologists, obstetricians, and vascular surgeons, in order to reduce the incidence of VTE and enhance the general medical care for VTE patients.

## Supplementary Material

Supplemental Material

## Data Availability

The data used and/or analysed in the context of the current study are available from the corresponding author upon reasonable request.
