# Supplementary material for: Hematologists’ awareness of venous thromboembolism in multiple myeloma: a national survey in China
Source: Ann Med. 2023 Nov 20;55(2):2263019. doi: 10.1080/07853890.2023.2263019 (PMC10836272; doi:10.1080/07853890.2023.2263019)
Supplement: Supplemental Material [file IANN_A_2263019_SM7290.zip › Supplementary file 1.docx]

**The Questionnaire** **(English translation)**

To note, the questionnaire sent to the participants was in Chinese. For the purpose of publication, it has been translated and exhibited in English.

1, How old are you?

○20-29 years old ○30-39 years old ○40-49 years old ○≥50 years old

2, What level of hospital do you work in?

○Township hospitals ○County hospitals ○Municipal hospitals ○Provincial or ministerial hospitals

3, How long have you been working?

○<5 years ○5-10 years ○11-20 years ○>20years

4, What’s your current professional title?

○Resident physician ○Attending physician ○Associate chief physician ○Chief physician

5, Please select the range of the annual admissions of patients with multiple myeloma (MM) by your medical group.

○5-10 ○11-20 ○21-40 ○41-80 ○>80

6, How much attention did you pay to the field of multiple myeloma?

○No (0 point) ○A little (1 point) ○Moderate (2 points) ○Much (3 points)

7, Which of the following conditions do you think could be classified as venous thromboembolism (VTE)? (multiple choice)

○Deep Vein thrombosis (DVT) ○Pulmonary embolism (PE) ○Superficial venous thrombosis ○Intramuscular venous thrombosis ○Myocardial infarction ○Cerebral infarction

8, How much attention did you pay to the field of MM-associated VTE?

○No (0 point) ○A little (1 point) ○Moderate (2 points) ○Much (3 points)

9, What is the incidence of superficial venous thrombosis in the MM patients you treated?

○<1% ○1-5% ○6-10% ○11-20% ○>20%

10, What is the incidence of DVT in the MM patients you treated?

○<1% ○1-5% ○6-10% ○11-20% ○>20%

11, What is the incidence of PE in the MM patients you treated?

○<1% ○1-5% ○6-10% ○11-20% ○>20%

12, Did you take the prothrombotic risk into account when choosing the treatment regimens for MM patients?

○No (0 point) ○Sometimes (1 point) ○Often (2 points) ○Always (3 points)

13, Did you choose different thromboprophylaxis for MM patients according to their treatment regimens?

○No (0 point) ○Sometimes (1 point) ○Often (2 points) ○Always (3 points)

14, Did you give thromboprophylaxis for MM patients with complications?

○No (0 point) ○Sometimes (1 point) ○Often (2 points) ○Always (3 points)

15, Did you implement VTE risk assessment for MM patients? What assessment tool did you choose?

○No assessment ○Caprini Score System ○Khorana Score System ○IMPEDE VTE Score System ○SAVED Score System○Self-made Score System

16, Did you carry out stratified thromboprophylaxis for MM patients?

○No stratification ○Stratified according to foreign guidelines ○Stratified according to clinical experience ○Stratified according to self-made guidelines

17, Did you implement bleeding risk assessment when carrying out thromboprophylaxis for MM patients?

○No (0 point) ○Sometimes (1 point) ○Often (2 points) ○Always (3 points)

18, Did you pay attention to the VTE symptoms when treating MM patients with IMiDs- dexamethasone-based regimen?

○No (0 point) ○Sometimes (1 point) ○Often (2 points) ○Always (3 points)

19, What do you think of the role of the following factors in VTE occurrence? You can evaluate each factor on a scale from 0 to 3 points, where a higher score represents a stronger prothrombotic effect.

○Chronic renal failure (requiring long-term catheterization and dialysis) ( )

○Chronic obstructive pulmonary disease (COPD) ( )

○Severe anemia ( )

○Immobility (for more than 72 hours) ( )

○Pathological fracture ( )

○Hypertension (very high risk group) ( )

○Chronic heart failure ( )

○Myocardial infarction ( )

○Antiphospholipid antibody syndrome ( )

○Peripherally inserted central catheter (PICC) ( )

○VTE history ( )

○Over the age of 70 ( )

○Use of erythropoietin stimulating agent (EPO) ( )

20, What thromboprophylaxis decision will you make for patients receiving VCd treatment without any additional risk factors?

○ Do not apply thromboprophylaxis ○ Aspirin ○ Warfarin○ LMWH ○ Rivaroxaban

21, What thromboprophylaxis decision will you make for patients receiving DVd treatment without any additional risk factors?

○ Do not apply thromboprophylaxis ○ Aspirin ○ Warfarin ○ LMWH ○ Rivaroxaban

22, What thromboprophylaxis decision will you make for patients receiving PAd treatment without any additional risk factors?

○ Do not apply thromboprophylaxis ○ Aspirin ○ Warfarin ○ LMWH ○ Rivaroxaban

23, What thromboprophylaxis decision will you make for patients receiving VRd treatment without any additional risk factors?

○ Do not apply thromboprophylaxis ○ Aspirin ○ Warfarin ○ LMWH ○ Rivaroxaban

24, What thromboprophylaxis decision will you make for patients receiving DRd treatment without any additional risk factors?

○ Do not apply thromboprophylaxis ○ Aspirin ○ Warfarin ○ LMWH ○ Rivaroxaban

25, What thromboprophylaxis decision will you make for patients receiving VTD-PACE treatment without any additional risk factors?

○ Do not apply thromboprophylaxis ○ Aspirin ○ Warfarin○ LMWH ○ Rivaroxaban

26, What thromboprophylaxis decision will you make for patients receiving KRd treatment without any additional risk factors?

○ Do not apply thromboprophylaxis ○ Aspirin ○ Warfarin○ LMWH ○ Rivaroxaban

27, What thromboprophylaxis decision will you make for MM patients who suffer a relapse and are treated with pomalidomide and dexamethasone?

○ Do not apply thromboprophylaxis ○ Aspirin ○ Warfarin○ LMWH ○ Rivaroxaban

28, What thromboprophylaxis decision will you make for MM patients who achieved a complete response (CR) after induction treatment and received lenalidomide (10mg/d) for maintenance therapy?

○ Do not apply thromboprophylaxis ○ Aspirin ○ Warfarin○ LMWH ○ Rivaroxaban

29, What medical decision will you make for MM patients who develop DVT during the third course of the VRd treatment with the response assessment as a partial response (PR)?

○ Anticoagulation and continue the original anti-MM treatment

○ Anticoagulation and substitute VRd with less thrombogenic agents

○ Anticoagulation and suspend treatment for primary disease

30, What medical decision will you make for MM patients who develop superficial venous thrombosis during the third course of the VRd treatment with the response assessment as a PR?

○ Anticoagulation and continue the original anti-MM treatment

○ Anticoagulation and substitute VRd with with less thrombogenic agents

○ Anticoagulation and suspend treatment for primary disease

Here are six clinical vignettes. Please select a thromboprophylaxis method for each vignette that you would most like to recommend. All patients in the vignettes have announced they would accept medical treatment decision. There is no wrong answer.

○ Do not apply thromboprophylaxis (0 point).

○ Aspirin (1 point);

○ Warfarin (INR 2.0-3.0; 2 points);

○ LMWH in prophylactic dose (such as enoxaparin 40 mg/d; 2 points);

○ LMWH in therapeutic dose (such as enoxaparin 1 mg/kg, twice daily; 3 points);

○ Rivaroxaban in prophylactic doses (10 mg/d; 2 points);

○ Rivaroxaban in therapeutic dose (20 mg/d; 3 points);

31, **Vignette 1:** A 76-year-old male was diagnosed with MM. He was assessed as complete response (CR) after induction treatment and received lenalidomide (10 mg/d) for maintenance therapy. What thromboprophylaxis decision will you make for him?

32, **Vignette 2:** A 78-year-old male patient has a history of smoking for more than 50 years and was diagnosed with COPD twenty years ago. He was hospitalized in the respiratory department because of a cough after a cold. The laboratory findings suggested a decrease in hemoglobin (70 g/L) and an increase in globulin (42 g/L).The CT scan showed multiple bone destruction in the ribs and some chronic infectious lesions in the lungs. The echocardiography indicated normal valve activity and ejection fraction. He was diagnosed with MM and transferred to the hematology department for further treatment. If the patient is treated with Rd, what thromboprophylaxis decision will you make for him?

33, **Vignette 3:** A 65-year-old male patient was newly diagnosed with MM. He has had hypertension for more than ten years and has been taking medicine irregularly. No significant abnormality was found on lung CT. The cardiac MRI suggested an anemic heart disease.The echocardiography showed a ventricular septum of 1.1 cm. The bone marrow and subcutaneous fat were negative for Congo red staining. If the patient is treated with VRD, what thromboprophylaxis decision will you make for him?

34, **Vignette 4:** A 65-year-old male patient visited the pain department because of bone pain for one week. He was sent to the hematology department due to elevated globulin and monoclonal immunoglobulinemia. He was then diagnosed with MM after related examinations. The patient had obvious bone pain and required bed rest. He had a PICC implanted due to poor vascular conditions. Will you choose the Rd regimen for him?

○ Avoid the IMiDs-based regimens ○ Choose the IMiDs-based regimens

If the patient is treated with Rd, what thromboprophylaxis decision will you make for him? (See options above)

35, **Vignette 5:** A 65-year-old male patient was newly diagnosed with MM. He experienced a thrombosis in the left common iliac vein ten years ago and has completely recovered without any sequelae. Will you choose the VRD regimen for him? If the patient is treated with VRD, what thromboprophylaxis decision will you make for him?

○ Avoid the IMiDs-based regimens ○ Choose the IMiDs-based regimens

If the patient is treated with Rd, what thromboprophylaxis decision will you make for him? (See options above)

36, **Vignette 6:** An 80-year-old female was diagnosed with pathological femur fracture due to an accidental fall. She was immobilized in plaster and bedridden for more than a week. The X-ray revealed multiple areas of bone destruction. The laboratory findings suggested a decrease in hemoglobin (85g/L) and an increase in globulin (85g/L). The serum and urine protein electrophoresis portrayed a monoclonal peak at IgG lambda, and the M-protein concentration was 56g/L. The diagnosis of MM was subsequently confirmed by bone marrow aspiration. Will you choose the Rd regimen for her? If the patient is treated with Rd, what thromboprophylaxis decision will you make for her?

○ Avoid the IMiDs-based regimens ○ Choose the IMiDs-based regimens

If the patient is treated with Rd, what thromboprophylaxis decision will you make for him? (See options above)
